# Comparative Transcriptome Analysis of Two Races of *Heterodera glycines* at Different Developmental Stages

**DOI:** 10.1371/journal.pone.0091634

**Published:** 2014-03-24

**Authors:** Gaofeng Wang, Deliang Peng, Bingli Gao, Wenkun Huang, Lingan Kong, Haibo Long, Huan Peng, Heng Jian

**Affiliations:** 1 The Key Laboratory for Biology of Insect Pests and Plant Disease, Institute of Plant Protection, Chinese Academy of Agricultural Sciences, Beijing, China; 2 Department of Plant Pathology, China Agricultural University, Beijing, China; 3 Huzhou Modem Agricultural Biotechnology Innovation Center, Shanghai Institutes for Biological Sciences, Chinese Academy of Sciences, Huzhou, Zhejiang, China; 4 Key Laboratory of Pests Comprehensive Governance for Tropical Crops, Environment and Plant protection Institute, Chinese Academy of Tropical Agricultural Science, Danzhou, China; James Hutton Institute, United Kingdom

## Abstract

The soybean cyst nematode, *Heterodera glycines*, is an important pest of soybeans. Although resistance is available against this nematode, selection for virulent races can occur, allowing the nematode to overcome the resistance of cultivars. There are abundant field populations, however, little is known about their genetic diversity. In order to elucidate the differences between races, we investigated the transcriptional diversity within race 3 and race 4 inbred lines during their compatible interactions with the soybean host Zhonghuang 13. Six different race-enriched cDNA libraries were constructed with limited nematode samples collected from the three sedentary stages, parasitic J2, J3 and J4 female, respectively. Among 689 putative race-enriched genes isolated from the six libraries with functional annotations, 92 were validated by quantitative RT-PCR (qRT-PCR), including eight putative effector encoding genes. Further race-enriched genes were validated within race 3 and race 4 during development in soybean roots. Gene Ontology (GO) analysis of all the race-enriched genes at J3 and J4 female stages showed that most of them functioned in metabolic processes. Relative transcript level analysis of 13 selected race-enriched genes at four developmental stages showed that the differences in their expression abundance took place at either one or more developmental stages. This is the first investigation into the transcript diversity of *H. glycines* races throughout their sedentary stages, increasing the understanding of the genetic diversity of *H. glycines*.

## Introduction

The soybean cyst nematode (SCN), *Heterodera glycines*, causes significant damage to soybean, resulting in annual yield losses between 1.9 and 3.5 million tons in the USA [1]. Developing resistant cultivars to SCN has proven to be a highly effective strategy to control this nematode [2]. Several resistance genes and QTLs conferring resistance to *H. glycines* have been identified [2]–[8]. However, few of these have been introgressed into commercially viable cultivars [2]. Field populations of *H. glycines* have been shown to have high variability [9], and the significant diversity of field populations combined with lack of applicable resistance sources could potentially lead to population shifts over time, resulting in virulent populations. For example, repeated growth of potato cultivars carrying the H1 resistance gene against *Globodera rostochiensis* has led to selection for *G. pallida* in parts of Europe [10].

Different phytonematode populations have different reproductive capabilities and other traits. For example, clumping behavior differs among root-knot nematode strains [11]. These differences suggest genetic diversity in phytonematode populations, and the same is likely to apply to SCN populations [12]. Investigating the genetic diversity of SCN populations will be helpful to recognize their population shifts and assist in control of them. However, there is insufficient research about the genetic diversity among SCN populations. Genetic studies that characterize *H. glycines* populations differing in their virulence have been carried out. RAPD-PCR was developed and employed for distinguishing two *H. glycines* populations with different virulence characteristics on soybean [13]. In addition, draft genome sequences of two *H. glycines* inbred avirulent and virulent lines were generated, and comparative genomic analysis was carried out [14]. These studies focused on differences in the genomes of *H. glycines* populations rather than differences in proteomes or transcriptomes. Soluble proteins of four SCN races were characterized and a 75 kDa protein was found to be unique to race 3 and race 4 as compared to race 1 and race 2 [15]. More recently, a comparative transcriptome analysis of two SCN inbred lines undergoing resistant and susceptible reactions yielded hundreds of differentially expressed genes, which provided some insights into the molecular mechanisms of diverse reproductive abilities between these two *H. glycines* lines [16]. However, there is no report on the transcriptional variation within SCN races across the full range of the parasitic stages or within the same sedentary stages.

In this study, we describe comparative transcriptome analysis between two SCN inbred lines, race 3 and race 4, at three parasitic stages, parasitic J2, J3 and J4 female, respectively. 92 race-enriched genes were identified in the two races at the three developmental stages. GO analysis of the race-enriched genes and gene expression profile analysis of 13 selected genes were also performed. Our results will increase the knowledge of the transcriptional variation between two SCN races.

## Materials and Methods

### Biological material

**Figure 1:**
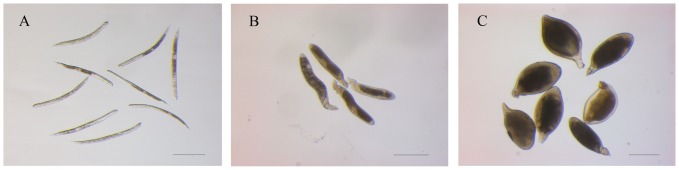
Morphology of *H. glycines* at different developmental stages. The sedentary *H. glycines* J2s, J3s and J4 females were isolated from soybean roots at 5 dpi, 7 dpi, and 12 dpi, respectively. The morphology of *H. glycines* at parasitic J2 stage (**A**), J3 stage (**B**) and J4 female stage (**C**) was examined with a microscope. Bar = 200 µm.

Two *H. glycines* inbred lines, race 3 and race 4, were propagated on conventional susceptible soybean genotype Zhonghuang 13 in a greenhouse at 28°C. To isolate different parasitic stages of *H. glycines*, one-week-old soybean plants were inoculated with preparasitic second stage juveniles (J2s). Infected roots were washed with sterile water to remove the uninfected nematodes from the roots 24 h post inoculation, then the infected soybeans were planted in sterile sandy soil (sand∶soil = 3∶1) [12]. The infected roots were harvested 5, 7 and 12 days post inoculation (dpi) for the collection of parasitic J2s, J3s and J4 females, respectively. The roots were homogenized for 30 seconds at medium speed in a kitchen blender, and the root homogenate was washed through successive 850, 250 and 25 µm sieves. Nematodes collected from the 25-µm sieve were purified by sucrose centrifugation [17], and the nematodes at different stages of development were hand-picked under a stereomicroscope (Figure 1).

### Suppression subtractive hybridization

The collected nematodes were homogenized in 1 mL TRIzol reagent and subjected to sonication for 3 minutes [18]. Total RNA was isolated according to the manufacturer's instructions (Invitrogen, USA). Total RNAs were resuspended in 3.5 µL of RNase-free water and mRNAs were amplified using the BD SMART mRNA Amplification Kit according to the manufacturer's recommendations (Clontech, USA). Firstly, nanogram quantities of total RNA, the cDNA Synthesis Primer II A, the SMARTT7 Oligonucleotide and MMLV Reverse Transcriptase were used to synthesize first-strand cDNAs with the T7 promoter at the 5′ end. Double-stranded cDNAs were generated with T7 Extension Primer and Advantage 2 PCR Kit (Clontech, USA). Following this, double-stranded cDNAs were purified with the NucleoSpin Extraction Kit (included in the BD SMART mRNA Amplification kit) and were transcribed into sense RNAs with T7 RNA Polymerase in *vitro* at 37°C for 12 h. Using the synthesized sense RNAs, double-stranded cDNAs were synthesized and amplified using the BD SMARTer Pico PCR cDNA Synthesis kit according to the manufacturer's recommendations (Clontech, USA). For the three life stages of *H. glycines*, parasitic J2s, J3s and J4 females, reciprocal Suppression Subtractive Hybridizations (SSH) were performed in both directions with the PCR-Select cDNA Subtraction Kit (Clontech, USA) according to the manufacturer's instructions. For each life stage, the forward (race 4 vs race 3) and reverse (race 3 vs race 4) subtractions were designed to select race 4-specific and race 3-specific cDNAs, respectively. In the forward subtraction, 117 ng of cDNA from race 4 was used as the tester and 572 ng of cDNA from race 3 as the driver. In the reverse subtraction, 117 ng of cDNA from race 3 was used as the tester and 572 ng of cDNA from race 4 as the driver. For each subtraction, two tester populations were created separately by ligating suppression adapter 1 or 2R to the blunt-ended *Rsa*I-digested cDNAs. The two tester populations were mixed with a 40-fold excess of drivers (the driver cDNAs had no adaptors) in separate tubes. The cDNA fragments specific to the tester were then amplified by a primary PCR consisting of 30 cycles with PCR primer 1 and a secondary PCR of 16 cycles using nested primers 1 and 2R. The amplified cDNA fragments were purified from the PCR mixture with TIANgel Midi Purfication kit (Tiangen, Beijing, China) according to the manufacturer's instructions.

### Construction of the subtraction libraries and sequence analysis

The cDNA fragments generated by the forward and reverse SSH were used to construct subtraction libraries. In each case, 30 ng of purified cDNA fragments was cloned into the pGEM-T Easy vector (Promega, USA). *Escherichia coli* cells (DH5α) were used to transform the resulting constructs by electroporation and plated on SOC medium supplemented with 5-bromo-4-chloro-3-indolyl-beta-Dgalactopyranoside (X-gal; 100 µg/mL) (Sigma, USA), isopropyl-beta-D-thiogalactopyranoside (IPTG; 60 µg/mL) (Sigma, USA) and ampicillin (100 µg/mL) (Sigma, USA). White colonies were picked and sequenced by BGI Tech (China). The sequences were trimmed to eliminate vector sequences, SMART-cDNA primers and SSH-adaptor sequences. The nematode cDNA sequences were then annotated for functional similarities in the GenBank database using the BLAST algorithms (http://www.ncbi.nlm.nih.gov/BLAST).

### Quantitative RT-PCR assay

Total RNAs were isolated from preparasitic J2s, parasitic J2s, J3s and J4 females using TRIzol regent according to the manufacturer's instructions (Invitrogen, USA). mRNAs were amplified using the BD SMART mRNA Amplification Kit (Clontech, USA) as described above. The mRNAs were then reverse-transcribed using oligo (dT)_20_ primers (Invitrogen, USA) and SuperScript III Reverse Transcriptase (Invitrogen, USA). qRT-PCR was performed on a Applied Biosystems 7500/7500 Fast Real-Time PCR System (Life technologies, USA) with 20-µL PCR mix containing 2 µL of cDNA, 200 nM of the appropriate oligonucleotide primers, 10 µL of 2× SYBR Premix Ex Taq and 0.4 µL of 50× ROX Reference Dye II (TαKαRα, Japan). The mix was preheated at 95°C for 15 seconds, followed by 40 cycles of 95°C for 3 seconds, 60°C for 30 seconds. Melting curve analysis was performed from incubation at 65°C followed by 0.5°C incremental ramp up to 95°C. Melting curve lines were used to validate product specificity. The *H. glycines* glyceraldehyde-3-phosphate dehydrogenase encoding gene (GAPDH, GenBank accession: CA939315) was used as an internal reference [19]. The primers used for GAPDH amplification and gene-specific primers used for qRT-PCR are listed in Table S1. Standard curve lines were established with four appropriate serial dilutions of first-strand cDNAs. Relative transcript abundance was calculated using the 2^−ΔΔCt^ method [20], and the cut-off was 2.0 [21]. qRT-PCR was carried out in triplicate for each of the two different samples from each nematode stage. For relative gene expression level analysis of race 3-enriched genes between race 3 and race 4, values were expressed as the increase levels relative to the calibration sample from race 4 at the same nematode developmental stage, and the reverse for that of race 4-enriched genes. For gene expression profile analysis, preparasitic J2 stage was considered as the calibration stage, and the gene expression level at this stage was defined as 1.0.

### Annotation of differentially expressed genes

Gene annotation was carried out using the BLAST2GO program [22]. NCBI-BLAST was carried out against database nr with Blastx and in database nt with Blastn with e-value cut-off of 1.0e-6 as well as the number of retrieved BLAST hits being 20. Annotation was done with default parameters.

## Results

### Race-enriched cDNA libraries of SCN race 3 and race 4 parasitic stages

Three race-enriched cDNA libraries were constructed for *H. glycines* race 3 and race 4 at three different sedentary stages (Figure 1). More than 300 clones from each library were sequenced.

359 sequences were obtained from the race 3-enriched parasitic stage J2 library, including 48 contigs and 52 singletons representing a total of 100 gene fragments. Similarly, 343 sequences were identified from race 4-enriched parasitic stage J2 library comprising 43 contigs and 58 singletons, representing 101 gene fragments. 58 and 67 gene fragments from the race 3-enriched parasitic stage J2 library and the race 4-enriched parasitic stage J2 library were found to have no homology with known genes in GenBank database, respectively (Table 1).

**Table 1:** Characteristics of race 3- and race 4-enriched cDNA libraries at three sedentary stages.

Library	ESTs^a^	Contigs	Uniques	Unigenes+^b^	Unigenes−c
Race 3 at parasitical J2 stage	359	48	52	43	58
Race 4 at parasitical J2 stage	343	43	58	34	67
Race 3 at J3 stage	446	51	264	195	120
Race 4 at J3 stage	579	86	261	157	190
Race 3 at J4 female stage	455	52	232	122	162
Race 4 at J4 female stage	492	57	256	139	174

a Total number of clones isolated from different race-enriched cDNA libraries at three sedentary stages.

b Unigenes with functional annotation.

c Unigenes without functional annotation.

The race 3-enriched J3 library yielded 446 sequences clustered into 51 contigs and 264 singletons, with a total of 315 gene fragments. For the race 4-enriched J3 library, 579 sequences were obtained and grouped into 86 contigs and 261 singletons, with a total of 347 gene fragments. There were 120 and 190 gene fragments from race 3 and race 4 with no match to known genes in GenBank, respectively (Table 1).

Finally, the race 3-enriched J4 female library gave 455 sequences, containing 52 contigs and 232 singletons, with a total of 284 gene fragments. The race 4-enriched J4 female library comprised 492 sequences that were clustered into 57 contigs and 256 singletons, corresponding to 313 gene fragments. 162 gene fragments from race 3 and 174 from race 4 had no match to known genes in GenBank (Table 1).

### More race-enriched genes were validated at later sedentary stages in both race 3 and race 4

Differentially regulated genes isolated from the race-enriched cDNA libraries were validated by qRT-PCR. In the parasitic J2 stage, there were seven race-enriched genes within race 3 and race 4, including six race 3-enriched genes and one race 4-enriched gene (Table S2). They contained three putative effectors, a cellulase (Hg3J2-CT16), a putative gland protein G28B03 (Hg3J2-07F02) and a pectate lyase (Hg4J2-04F08) (Table S2). At the J3 stage, the number of race-enriched genes was increased to 29 with 20 race 3-enriched genes that included a cellulose binding protein (Hg4J3-11E01) and nine race 4-enriched genes (Table S2). At the J4 female stage, the number reached 56, of which 43 race-enriched genes were identified in race 3, and 13 in race 4 including three putative effectors, a chorismate mutase (Hg4J4-09D03), a putative gland protein G11A06 (Hg4J4-CT26) and CLAVATA3/ESR(CLE)-related protein 2 (Hg4J4-CT33) (Table S2). The total numbers of race-enriched genes at parasitic J2 stage, J3 stage and J4 female stage was 7, 29 and 56, respectively, indicating that more race-enriched genes were identified with the development of race 3 and race 4 in soybean roots.

### Race-enriched genes are significantly enriched for GO category ‘metabolic process’

**Figure 2:**
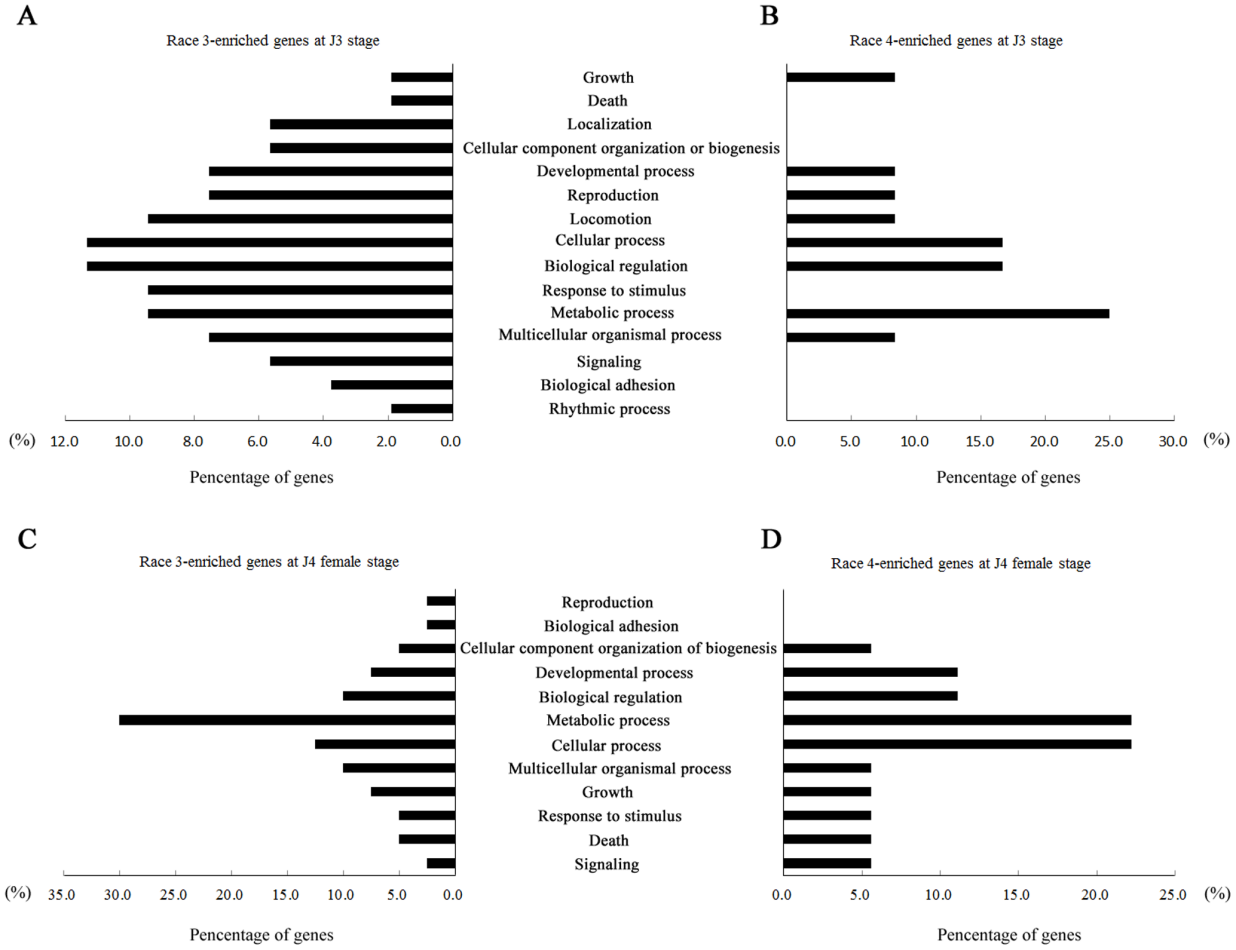
Gene Ontology analysis of race-enriched genes at sedentary stages J3 and J4 female. The gene annotation of induced genes found in race 3 (**A**) and race 4 (**B**) at J3 stage, and the ones in race 3 (**C**) and race 4 (**D**) at J4 female stage was carried out with BLAST2GO. The horizontal number represents the percentage of genes involved in different GO categories in all the induced genes found in both the two races at J3 and J4 stages, respectively.

GO analysis of all the race-enriched genes in both race 3 and race 4 at J3 and J4 female stages were conducted, respectively. At sedentary stage J3, for race 3, the top five gene-enriched GO categories were ‘cellular process’ (11.3%), ‘biological regulation’ (11.3%), ‘locomotion’ (9.4%), ‘response to stimulus’ (9.4%) and ‘metabolic process’ (9.4%) (Figure 2A). For race 4, the top three were ‘metabolic process’ (25.0%), ‘cellular process’ (16.7%) and ‘biological regulation’ (16.7%) (Figure 2B). At sedentary stage J4 female, for race 3, the top four were ‘metabolic process’ (30.0%), ‘cellular process’ (12.5%) and ‘biological regulation’ (10.0%) as well as ‘multicellular organismal process’ (10.0%) (Figure 2C). In the case of race 4, the top four were ‘metabolic process’ (22.2%), ‘cellular process’ (22.2%), ‘developmental process’ (11.1%) and ‘biological regulation (11.1%) (Figure 2D). These data revealed that the most of race-enriched genes were clustered into the GO category ‘metabolic process’.

### Transcript abundance of 13 race-enriched genes at four developmental stages

To further investigate the transcript abundance of race 3- or 4-enriched genes, 13 differentially expressed genes including eight putative effector coding genes were selected for detailed analysis of their relative expression levels at preparasitic J2, parasitic J2, J3 and J4 female stages in *H. glycines* race 3 and race 4, respectively. Compared to that of race 4, the race 3-enriched genes, such as the cellulase gene (Hg3J2-CT16), putative gland protein G28B03 coding gene (Hg3J2-07F02), and cellulose binding protein gene (Hg4J3-11E01), had 2.3 to 2.9 fold higher expression levels at the parasitic J2 stage, while the 14-3-3 encoding gene (Hg3J4-02E05) showed 131.6 and 45.1 fold higher expression level at J3 and J4 female stages, respectively (Table 2). Compared to that of race 3, race 4-enriched genes, the pectate lyase gene (Hg4J2-04F08) had 3.4 fold more transcripts at the parasitic J2 stage, while three other effectors including CLAVATA3/ESR (CLE)-related protein 2 (Hg4J4-CT33), putative gland protein G11A06 (Hg4J4-CT26) and chorismate mutase (Hg4J4-09D03) coding genes had 453.2, 20.0 and 58.7 fold higher expression levels at J4 female stage, respectively (Table 3). In contrast, there were no obvious expression level changes for the three genes at preparsitic J2, parasitic J2 and J3 stages (Table 3).

**Table 2:** Relative abundance of race 3-enriched genes at four different stages in race 3 compared with that in race 4.

		Relative abundance^a^			
Gene ID	Best hit description	pi-J2	p-J2	J3	J4
Hg3J2-CT16	Cellulase	1.5	2.3	0.7	0.2
Hg3J2-07F02	Putative gland protein G28B03	1.9	2.9	0.7	0.0^b^
Hg3J3-07B01	Aspartic protease precursor Hgg-33	0.7	1.1	2.1	0.7
Hg4J3-07C01	Ubiquitin-conjugating enzyme e2, putative	1.2	0.8	2.5	1.0
Hg4J3-11E01	Cellulose binding protein	3.3	2.9	2.0	1.8
Hg3J4-02E05	14-3-3 protein	1.6	0.8	131.6	45.1
Hg3J4-CT26	Polyubiquitin	1.1	1.7	1.4	2.2

a The expression level of genes was analyzed with the 2^−ΔΔCt^ method using the GAPDH gene as an internal reference gene for normalization. The calibrator was the transcript level of the corresponding gene in race 4 at the same developmental stage.

b The relative abundance of the gene indicated was very close to zero.

**Table 3:** Relative abundance of race 4-enriched genes at four different stages in race 4 compared with that in race 3.

		Relative abundance^a^			
Gene ID	Best hit description	pi-J2	p-J2	J3	J4
Hg4J2-04F08	Pectatelyase (pel-4) mRNA, complete cds	0.7	3.4	0.6	0.2
Hg4J4-CT33	CLAVATA3/ESR (CLE)-related protein 2	0.8	0.7	1.9	453.2
Hg4J4-CT26	Putative gland protein G11A06	0.8	0.5	1.2	20.0
Hg4J4-09D03	Chorismate mutase	1.1	0.7	1.4	58.7
Hg4J4-09C10	Glutathione S-transferase-1	2.9	3.5	3.2	10.9
Hg4J4-05D07	Mannitol dehydrogenase domain protein	0.0^b^	1.6	2.5	8.7

a The expression level of genes was analyzed with the 2^−ΔΔCt^ method using the GAPDH gene as an internal reference gene for normalization. The calibrator was the transcript level of the corresponding gene in race 3 at the same developmental stage.

b The relative abundance of the gene indicated was very close to zero.

### Expression profiles of race-enriched genes between race 3 and race 4

**Figure 3:**
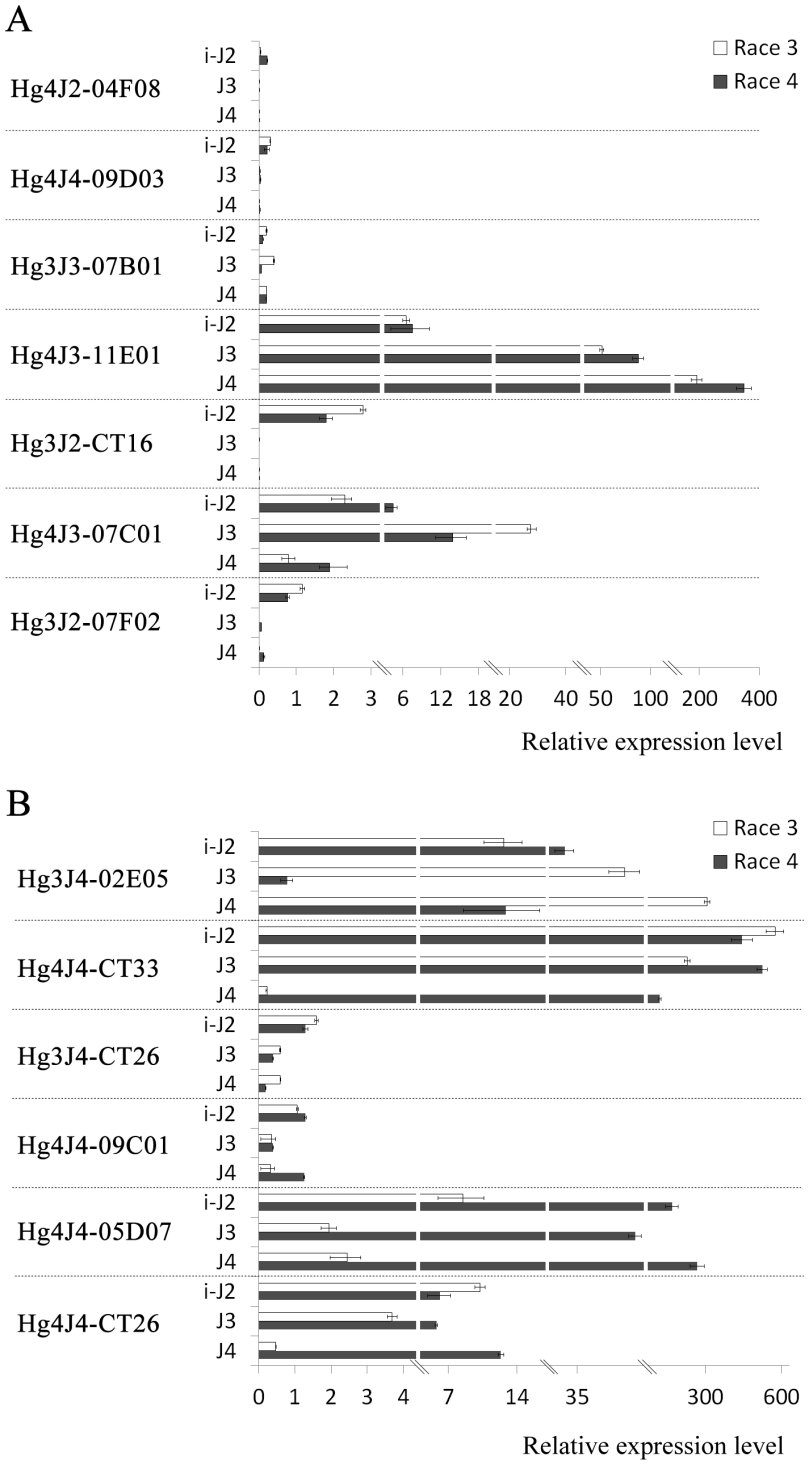
Gene expression profiles of 13 race-enriched genes in both race 3 and race 4. RNA samples were isolated from preparasitic J2s, parasitic J2s (i-J2), J3s (J3) and J4 females (J4 female) of SCN race 3 and race 4, respectively. The expression profiles of the 13 genes categorized into similar (**A**) and uncoordinated (**B**) expression profiles in both the races. Preparasitic J2 stage was considered as the calibration stage, and the expression level of these 13 genes at preparasitic J2 stage was arbitrarily set to 1.0. The expression level was analyzed with the 2^−ΔΔCt^ method using the GAPDH gene as an internal reference gene for normalization. Mean and standard errors were determined with data from three independent replicates.

In order to elucidate the expression profiles of the above genes (Table 2, 3), we assayed their relative expression levels at parasitic J2, J3 and J4 female stages compared with that at preparasitic J2 stage within race 3 and race 4, respectively. The results revealed that seven selected genes had similar expression tendencies in race 3 and race 4 (Figure 3A). Hg4J2-04F08 and Hg4J4-09D03 in both the races were all sharply down-regulated at parasitic J2 and J3 stages, and had nearly no detectable transcripts at J4 female stage, while Hg3J3-07B01 was significantly down-regulated at parasitic J2 stage but with no obvious change in its expression level at the subsequent later stages in both race 3 and race 4 (Figure 3A). In contrast, the expression of Hg4J3-11E01 went up throughout the parasitic stages in both the races (Figure 3A). The transcript levels of Hg3J2-CT16 were increased at sedentary J2 stage and reduced to undetectable level at both the following J3 and J4 female stages in the two races (Figure 3A). Hg4J3-07C01 was up-regulated with the development of nematodes except for J4 female stage with down-regulation in race 3 and race 4 (Figure 3A). Transcript levels for Hg3J2-07F02 were not changed notably at parasitic J2 stage, but went down significantly at J3 stage and kept the low levels at J4 female stage in race 3 and race 4 (Figure 3A).

Interestingly, six genes including Hg3J4-02E05, Hg4J4-CT33, Hg3J4-CT26, Hg4J4-09C10, Hg4J4-05D07, and Hg4J4-CT26 had uncoordinated expression profiles (Figure 3B). Diversities of gene expression patterns for Hg3J4-02E05 and Hg4J4-CT33 between race 3 and race 4 were both observed at J3 stage (Figure 3B). The expression of Hg3J4-02E05 rose throughout the sedentary stages, J2, J3 and J4 female in race 3, while it went up at parasitic J2 stage and went down at J3 stage followed by a remarkable increase at the J4 female stage in race 4 (Figure 3B). For Hg4J4-CT33, expression went up at sedentary J2 stage in race 3 and race 4, while it went down notably in race 3 but showed no change in race 4 at J3 stage followed by a sharp down-regulation at J4 female stage in both the races (Figure 3B). Similarly, the expression profiles of Hg3J4-CT26, Hg4J4-09C10, and Hg4J4-05D07 differed only at the J4 stage between the two races. The expression of Hg3J4-CT26 was similar until parasitic J2 stage and decreased at J3 stage in race 3 and race 4, then showed different patterns at the J4 female stage with similar transcript abundance in race 3 but continuous reduction in race 4 as compared with that at J3 stage (Figure 3B). Hg4J4-09C10 shared a similar expression pattern with that of Hg3J4-CT26 at parasitic J2, and J3 stages in both the races, however, its transcript abundance at J4 female stage was nearly unchanged in race 3 but was sharply enhanced in race 4 (Figure 3B). Hg4J4-05D07 was up-regulated at sedentary J2 stage, and down-regulated at J3 stage in both the races, however, its expression level at J4 female stage was almost unaltered in race 3 but was significantly increased in race 4 (Figure 3B). Unlike the expression profiles of above genes, Hg4J4-CT26 went up at parasitic J2 stage in race 3 and race 4, however, its expression tendency was totally different in the following stages with a decline at subsequent J3 and J4 female stages in race 3 and nearly unchangeable expression level at J3 stage and a dramatic increase at J4 female stage in race 4 (Figure 3B), which suggested that the expression profiles of Hg4J4-CT26 differed at both J3 and J4 female stages between the two races.

## Discussion

Although there are 16 races in soybean cyst nematode [23], [24], limited information on transcriptional differences between the races is available. Investigation of the differences in transcriptomes between SCN races will be helpful to understand the various nematode-host interactions. Transcriptional diversities of two SCN inbred lines have been characterized, but this analysis was limited to the first eight days in the incompatible interaction [16]. In order to overcome this limitation, two *H. glycines* inbred lines, race 3 and race 4, and their susceptible host soybean cultivar Zhonghuang 13 were used to allow an examination of the transcriptional differences within the two races throughout the sedentary stages. Moreover, the materials used, namely parasitic J2s, J3s and J4 females, were manually microdissected to guarantee the nematodes being at the same developmental stages (Figure 1), which could avoid data distortion resulted from the non-synchronized development of nematodes in host roots.

In this study, a total of 92 race-enriched genes within race 3 and race 4 were identified at the three parasitic stages tested, including several putative effectors including a cellulase encoding gene (Hg3J2-CT16) and a putative gland protein G28B03 encoding gene (Hg3J2-07F02) (Table S2). In addition, among the race-enriched genes, there were several other genes similar to sequences reported as being present in the secretomes of *Meloidogyne incognita* and *Bursaphelenchus xylophilus* [25], [26], including unc-87 (Hg3J2-CT20), ubiquitin-conjugating enzyme e2 (Hg4J3-07C01), serine carboxypeptidase (Hg3J4-07H09), serine proteinase (Hg3J4-CT10), fructose-1,6-bisphosphatase 1 (Hg4J4-06C04) and hypothetical protein bm1_50160 (Hg4J3-05D07) (Table S2). Interestingly, more race-enriched genes were identified in race 3 than that in race 4 at all the three sedentary stages examined, and the numbers of race-enriched genes, for the two races, were also increased with the development of them in soybean roots (Table S2). A similar result was also observed in a previous study [16]. GO analysis of the race-enriched genes showed that most functioned in metabolic process (Figure 2). Based on the above data, it might indicate that there was an increase in transcriptional diversity between race 3 and race 4 with their development in soybean roots, and genes involved in metabolic process were one of the main contributionors to these differences. Feeding sites induced by phytonematodes are thought to be metabolically highly active [27], and act as a nutrient sink providing nutrients for phytonematode survival and development [28]. It is unclear whether there is a relationship between the diverse transcript abundance of the race-enriched genes involved in metabolic processes and the nematode nutrient feeding ability for both race 3 and race 4.

Relative transcript level analysis of the 13 selected race-enriched genes showed that these genes can be differentially expressed at either one or more developmental stages (Table 2, 3). The CLAVATA3/ESR (CLE)-related protein 2 encoding gene (Hg4J4-CT33) was highly expressed in race 4 J4 females (Figure 3B) and its transcript abundance was more than 400 fold higher in race 4 J4 females compared with that in race 3 J4 females (Table 3). CLE genes were found to mimic plant CLE to modulate plant development and host defense to facilate nematode infection and feeding site formation and maintenance [29], [30]. Moreover, the CLE variable domain might play an important role in host-specific recognition [31]. Interestingly, we found that only one residue was different between the CLE gene cloned from *H. glycines* race 4 (Hg4J4-CT33) and the one isolated from *H. glycines* previously (GenBank accession: Q86RQ1) in variable domain II (unpublished data). The biological meaning of the sequence variation for both the CLE genes needs further research. Three genes (Hg4J3-11E01, Hg4J4-09C10 and Hg4J4-05D07) showed obvious different gene abundance at preparasitic J2 stage (Table 2, 3). These data are in agreement with the previous view that the variations in transcriptional activity prior to infestation exist between different *H. glycines* populations [16].

The gene developmental expression analysis of the 13 selected genes above was also carried out (Figure 3). The gene expression patterns of cellulase coding gene (Hg3J2-CT16) and putative gland protein G28B03 coding gene (Hg3J2-07F02) were in agreement with those observed in previous studies [32], [33], but not for aspartic protease precursor Hgg-33 encoding gene (Hg3J3-07B01) which went down at parasitic J2 stages in race 3 and race 4 [32] and putative gland protein G11A06 coding gene (Hg4J4-CT26) which went up at J4 female stage in race 4 [32]. In addition, the CLAVATA3/ESR(CLE)-related protein 2 coding gene (Hg4J4-CT33) was sharply down-regulated in race 3 J3s and significantly up-regulated in race 4 J4 females [31]. The others have not previously been assessed in SCN.

Six race-enriched genes, including the three putative effectors CLAVATA3/ESR (CLE)-related protein 2 coding gene (Hg4J4-CT33), putative gland protein G11A06 coding gene (Hg4J4-CT26) and glutathione S-transferase-1 coding gene (Hg4J4-09C10), had diverse expression tendencies between race 3 and race 4, and the differences occurred at J3 and J4 female stages (Figure 3B). According to the gene expression profiles of three effectors within race 3 and race 4, it was found that Hg4J4-CT26 played role at the three sedentary stages, parasitic J2, J3 and J4 female in race 4 but just at parasitic J2 and J3 stages in race 3 (Figure 3B). This was also the case for Hg4J4-CT33 (Figure 3B), while Hg4J4-09C10 functioned at parasitic J2 and J4 female stages in race 4 but just at parasitic J2 stage in race 3 (Figure 3B). These data suggest that all the three effectors were required for a longer time at sedentary stages in race 4 than that in race 3. Furthermore, all the three genes had higher transcript levels in race 4 than that in race 3 at one or more sedentary stages (Table 3).

Our data elucidated the transcriptional diversities between *H. glycines* race 3 and race 4 at the three different sedentary stages that representing the full diversity of parasitic stages present during nematode-host interaction [16]. This is the first investigation into the transcriptional variation between two SCN races throughout the sedentary stages. The various developmental expressions of six race-enriched genes within race 3 and race 4 is also a novel observation. However, the limitations of the SSH method (low sensitivity and low-throughput characterizations), and the very limited nematode materials available for this study resulted in fewer race-enriched genes being identified than expected, implying unavoidable loss of other potentially interesting genes. Maier *et al* have recently established a new method for RNA-seq analysis with strictly limited numbers of nematode gland cells, making it possible to conduct high-throughput analysis of the transcriptional diversities between two SCN races at all the sedentary stages [34]. In the future, using partially resistant hosts combined with this new method will allow changes in these expression profiles related to differences in the ability of the nematodes to develop on these hosts to be characterized in more detail.

## Supporting Information

Table S1 Primers used in qRT-PCR analysis.
(DOC)

Table S2 Validation of race-enriched genes within race 3 and race 4 at three parasitic stages by qRT-PCR.
(DOC)
