# Supplementary material for: Comparative Transcriptome Analysis of Two Races of Heterodera glycines at Different Developmental Stages
Source: PLoS One. 2014 Mar 24;9(3):e91634. doi: 10.1371/journal.pone.0091634 (PMC3963861; doi:10.1371/journal.pone.0091634)
Supplement: Table S1 — Primers used in qRT-PCR analysis. (DOC) [file pone.0091634.s001.doc]

**Table S1. Primers used in qRT-PCR analysis**

| **Genes** | **Primer sequences (5′-3′)** |
| --- | --- |
| GAPDH | TCCAAGGCATAGAAAGACGACG |
|  | AACAAGTCATTGGACGGCATCA |
| Hg3J2-07F02 | TATCCGAAGCCAATCCCGAGTC |
|  | TTTCTTTAGGCCATCGAATTTGTAC |
| Hg3J2-CT20 | CGATCAGCTCCTCCTTTTCCTCT |
|  | GAGCCGAATTGAAAATCCAAACCA |
| Hg3J2-CT24 | TCAGGGGCAAATAGAAAGGATGG |
|  | GCAGTTAAATTGGGCTCACAGAG |
| Hg3J2-CT48 | TAGTTGGCTCATTGCTTCTGTGG |
|  | CGGACATCTAAGGGCATCAC |
| Hg3J2-CT16 | CTCATCGGTGTAAACCAGAAGCC |
|  | ATGCTAAAGATGAAATGACCCAGA |
| Hg4J2-CT26 | GGGACGTATTCAGAGCGAGTTG |
|  | CGTGATTGGGATCGGACATTGC |
| Hg4J2-04F08 | AGCAGCAGCGAATGCAAGGA |
|  | TGATCGTCAACGTGCCACCG |
| Hg3J3-CT5 | TTGGTCTTTTGCCTGCCGAACG |
|  | TTGTGCCGTCCTCTGTCTGAAT |
| Hg3J3-01B03 | CGGTTTTATCAGTGCTGCAAATTGG |
|  | CTTCGAATGCCATTTCCGTCTCC |
| Hg3J3-01C12 | GCCTTTGGGCGTGCCATTCTTC |
|  | TTGGGATTGTTGTTGTTGCTGTGC |
| Hg3J3-01G05 | CACATAGCCCAACGATTCCACA |
|  | CACATTCCATACAGGGACCACAA |
| Hg3J3-CT1 | CATTTCGCAGTTTCCGTCTCCA |
|  | CACCAAATAGAAGGGTCCGAGT |
| Hg3J3-04C09 | GGAAAACCAACACCGGGACATT |
|  | ACGCCACTTTGTTCGTTGCTCT |
| Hg3J3-04D05 | CCAAATGTGCCTTGAAACAATCGC |
|  | CCAGGACGGAGTGCCAGAAA |
| Hg3J3-05C05 | AACAAAGGGCACGCCAGCCGAAG |
|  | CAGTAACGGCGCAAATGCGTAAGC |
| Hg3J3-07A01 | TTGCGAATTGCCGACTCGTACA |
|  | TGCGATCACAAGGCCTCCGTTA |
| Hg3J3-07B01 | CGGGTGTCGGAGAACTTTGGAT |
|  | CAACACGACTTTGGCCCACATC |
| Hg3J3-07C05 | TCTGAAGGCATCTGTATTTCTCGG |
|  | TGAGTCCGGCTTCGACCATTGT |
| Hg3J3-07E10 | ACCTCCGAGCAACCATTGACGC |
|  | GAACAGGCGGAACTTCAAACGG |
| Hg3J3-06H09 | GGAACAGATCGGAGTGACCAGC |
|  | TTTGCCAACCATTCGCAGTAGATA |
| Hg3J3-07B03 | AGTGCCATTGCGTTGCCCATCT |
|  | GCATTTGCCCTTCTCCAACTCC |
| Hg3J3-10F10 | GATGGGTAAATCTTTCGGGAAT |
|  | CTCTCCAACAGCCAACTCACTC |
| Hg4J3-05D07 | AACGCAACACTAAGCCCGACGAT |
|  | GTTGATTTCTCTGAACAGTGACGC |
| Hg4J3-07C01 | AACGGGTAGTCCCTCGGAAATG |
|  | ACGGCCACTTGATCAACGGACC |
| Hg4J3-CT55 | CGTAAATCAGCACGCCAATGTC |
|  | GTTGACGCATCACTAACGGACTG |
| Hg4J3-11B08 | AGAGCGGACGAGCAGAAGGAAT |
|  | CCAAAATGCTGGCCAAACATCTCC |
| Hg4J3-11B09 | CACCAGCAGTTGTGCCTTCAGT |
|  | GCGGCTTCGGCTCGTGTTTGTA |
| Hg4J3-11E01 | AGGCTGTCCATTTCCGCTCAAG |
|  | GAAAATGACTGCCGTGCCGAAC |
| Hg4J3-06F09 | CGGTGGATAATGCGGCAACTGA |
|  | GCGAAGGTGACAAAGCCGAATC |
| Hg4J3-07G08 | TTCCGCATCTTCTCCGTATTGT |
|  | ATGGTCAGATTCATTCCGTTGG |
| Hg4J3-08F06 | CCATTAATGGACTTTTTTTTTGGTC |
|  | TGAAAACCAGAGGAAAAAGCAACC |
| Hg4J3-08G05 | AGAGGGCGGGAGTCAAGCACAT |
|  | GTCCACTTGAAAGTCCACCATTG |
| Hg4J3-09E10 | AACCCACCGGGAACGATGTCTT |
|  | CCAACCACCAGAGTCGCAAAGG |
| Hg4J3-10H04 | TATTGCTTCGGTGGCTGCTGTT |
|  | TTTGCGCAAGATTGACGAGAACC |
| Hg4J3-CT31 | TGACGAGAAAAGCGACGAATGG |
|  | TTAACGAGCAGGGCACTGAGGC |
| Hg4J3-CT80 | CCACAGAAGCAATGAGCCAACTAT |
|  | GGGCATCACAGACCTGTTATTG |
| Hg4J3-CT16 | GGATGCGGGTGTAATGTCTTTGAA |
|  | GTCGACTGCACTGTTTTCTTTGG |
| Hg3J4-01G02 | CCGTCTCATTTTCTTATTGGTCGTG |
|  | GACATACAGCCGAAGTCCATCC |
| Hg3J4-01H11 | GCCTCGAACCATTGCACAGACT |
|  | AGACCTCGTCAAATGCCAAACTG |
| Hg3J4-02A03 | TGCCCCAAATACTGTGCCAACG |
|  | ATTTCGTCTTTTTATGCTCGTCGTG |
| Hg3J4-02E05 | GGCGATTTCTACCGAGACCAGG |
|  | GTTTCAACTTTGTGGTGGCAATGGT |
| Hg3J4-02H09 | AAATGCCAAAAATGAGAGTGCCAGT |
|  | TGGTCTGTCCAACAATCATCGTC |
| Hg3J4-04A02 | TGAATCCGGAAAAGCGGTGCAC |
|  | TGTTTTTGCTTAGCTTCTGCTTCGT |
| Hg3J4-04G03 | AACGGGTCCTTCGCCACGCATT |
|  | GTTGTTGTAATTGACTCCATTTGGT |
| Hg3J4-05D11 | AGACGCCGGAGAAACAATGACG |
|  | GACCACAGCACGAACAGCCACT |
| Hg3J4-06B04 | AGAGGGCGGAGTTGGGCACATT |
|  | AGTGGGCAGTGTTAGGTTGGCTT |
| Hg3J4-06B10 | CGAAAGGGATTTGGTGGTGCTG |
|  | AAAAGCCTTCGGTCGGTTGGTC |
| Hg3J4-06E03 | TCAGCCGACAAAAGTGACGAGC |
|  | GCGCATTGAATTAAACAAGCAGC |
| Hg3J4-06F04 | TTTCCACCTTTGTTGTATGTCTCTG |
|  | GCCAATCATTCATTTCACTCTGTTC |
| Hg3J4-06G09 | TTTCCCCTCCATAAATGCTTCGAT |
|  | CGGGCAGGTACTTTTTTTGACAG |
| Hg3J4-07C06 | TGATCCCTGGCACTGCGGTGTT |
|  | TCTGTCTCCGCCCCATCGCAT |
| Hg3J4-07C08 | ACTCTTTCTGCTTCCGTGCGATA |
|  | TTTCCCTTGCCATTGGATACGC |
| Hg3J4-07H09 | TGTCGCCGTAGAACAGCAGAACT |
|  | CAATTACAAGACACAATACGAGG |
| Hg3J4-09B07 | CGGGTAGCAGATGAAGCGGACA |
|  | GACCTGGCGAAGCAACCACTGAA |
| Hg3J4-09E07 | TAGTTGATTCGGCAGGTGAGTTGT |
|  | TCATTAGACCCCATAAAAGGTGTTG |
| Hg3J4-09E09 | GGCGATTTGGCAGAACGATTGA |
|  | TGGGATGGCTTTAATTTGGGTTC |
| Hg3J4-09G01 | AACAATGCCTTGGTTTGCTCGTC |
|  | ATGGTGGTCATTGAGTTTGAAGCA |
| Hg3J4-CT10 | GACGGCATTATGGAGTCGGAAGT |
|  | GACCAACAACGGACCACCACTG |
| Hg3J4-CT13 | CCGTCCGTCAGTCAAAATGCCAA |
|  | TCGTGTTCGTTGTTTGTGGCTCA |
| Hg3J4-CT17 | GGAGTAACGGATGGACAGTAAGCA |
|  | ATCATTTTTGAGTGTCGCAACCAG |
| Hg3J4-CT2 | GGCAAGCCATTCAAGGCATCAA |
|  | ATGTCAAGGGCGACGGAGAAGC |
| Hg3J4-CT21 | AAGCTCCAAAGTAAGTGAACAC |
|  | AATGACTCTGGCAAATACGTTCTG |
| Hg3J4-CT28 | GCGTGGCAAAGCATAAAAGGAC |
|  | TTCGGCTGCGTTCATTTGTTGC |
| Hg3J4-CT30 | AACGCCTGGGTTCAGAGTCAAG |
|  | CGGTAAAGCGAATGGTTAGAGG |
| Hg3J4-CT32 | GCTCTTTCGCGATGGCACTCAA |
|  | CGCTTTATGTCTTCTTCTCGTTTCT |
| Hg3J4-CT35 | GTTCAGCCAGCAGACAGGACAT |
|  | TTGACTTGTTCCCGGGGTTGAC |
| Hg3J4-CT36 | CCCGTAAATTTGGTCAATCAGTGG |
|  | TACAGCATCGAGGCGAACAACG |
| Hg3J4-CT48 | ACCACTTTGTATGTGCCTTTGATTG |
|  | ACAATCGGAAACGAAGAGTGCC |
| Hg3J4-CT54 | TATCAACACCCTCATTTGCTGCTT |
|  | TCCTCAACTTGCTCAGCGTCAG |
| Hg3J4-CT55 | ATTCGATGCATGAGGAAGAAGCTG |
|  | ATTGGCAGGACGATGACTTTGTG |
| Hg3J4-CT26 | ATTCCTTCTGGATGTTGTAGTCGG |
|  | ATCTTCGCTGGGAAACAACTCG |
| Hg3J4-CT39 | GCCTGCTGCCATCCTTAGACGT |
|  | TGGTGATGACGGGTAACGGAGG |
| Hg4J4-CT42 | GCATATCAGTAAGCGGAGGAAAAGA |
|  | TCCCAGTTCACTCGCCGTTA |
| Hg4J4-02C07 | GGGTCTAATGAGCGGGCACTT |
|  | GAACTGGCGATGTGGGATGA |
| Hg4J4-02E07 | CGGTTTCTATTTGATGTTGGTTGG |
|  | ATTACCCGTGCCCACAGCGAT |
| Hg4J4-03C06 | CCGACAACAACTTCCACCCAAT |
|  | ACAAAGGACCGACGCGAAA |
| Hg4J4-05D02 | GGTGGGATTGGAGCAGTGATTG |
|  | GAATTGTGCCCCTTGGTCATC |
| Hg4J4-06C04 | GACATACAGCCGAAGTCCATCC |
|  | GTATTCCAGCAATTCCTCCACGT |
| Hg4J4-06F02 | AGCACTTGAAGGTCCGTCGCAT |
|  | TGGCATGGATTGATGTCCGACT |
| Hg4J4-01F11 | CTGTTGCCAAGCATAAAGCCATA |
|  | AGGCCGTTTTCGATGATGTTG |
| Hg4J4-05D07 | CGACCGTTTGGCTGTCCAT |
|  | GACACGAAATGCGTCTGATTGG |
| Hg4J4-06A04 | AGCACGGACACTGCGACCATT |
|  | CTTCCCAATGCTCAATAGCCAGG |
| Hg4J4-06B03 | CTCCGACTTTGACCGATTGTTAC |
|  | AGGAATTGGGCCTGTTTAGTCTC |
| Hg4J4-06D05 | CTTCGTCAAATGGTGGCAATCC |
|  | CTCACAGCAAATTTCGGTGGATG |
| Hg4J4-06G09 | GGCTAAACAGGGAGGGCATC |
|  | AAGCAGCGGAAGTCGGCAAG |
| Hg4J4-07E04 | TTGTATTAGCTGAATCCAATCGTT |
|  | CCACTAACTAATTCTCTCTCACCTTC |
| Hg4J4-07F07 | CCAACGCCAACAGCAGCAGAA |
|  | CAATTGTGCGTCTGAACAGTCCA |
| Hg4J4-09C10 | TGGCGGAATCCCATTTTGACG |
|  | GGGAAGGCGAAGGCTAACGAA |
| Hg4J4-09D03 | TTTTCCACAGATGCCCAACAAT |
|  | AGATACCCGGCCCAAAGTGA |
| Hg4J4-CT26 | TGCCTGCCATTCGTTGTTGTTGC |
|  | ATGTTCGCCATTGCCGCCTCTG |
| Hg4J4-CT33 | CATCCATTCTTTCTTCCTCCGTTAG |
|  | GACCCGACCCACATCATCATTAG |
| Hg4J4-CT53 | GAAGGGCCTCAAGGACAGAATC |
|  | TGCTGACTGAGCCTTGGTGAGA |
