# Supplementary material for: Comparative Transcriptome Analysis of Two Races of Heterodera glycines at Different Developmental Stages
Source: PLoS One. 2014 Mar 24;9(3):e91634. doi: 10.1371/journal.pone.0091634 (PMC3963861; doi:10.1371/journal.pone.0091634)
Supplement: Table S2 — Validation of race-enriched genes within race 3 and race 4 at three parasitic stages by qRT-PCR. (DOC) [file pone.0091634.s002.doc]

**Table S2. Validation of race-enriched genes within race 3 and race 4 at three parasitic stages by qRT-PCR**

| **SCN stage** | **Gene ID** | **GenBank accession** | **Size (bp)** | **Best hit description** | **Best hit GenBank accession** | **E-value** | **Best hit organism** | **Fold change^a^** |
| --- | --- | --- | --- | --- | --- | --- | --- | --- |
| Race 3 at parasitic J2 stage | Hg3J2-07F02 | JZ532492 | 654 | Putative gland protein G28B03 | AAP30764 | 3.00E-94 | *Heterodera glycines* | 2.5 |
|  | Hg3J2-CT20 | JZ532493 | 170 | Unc-87 (unc87) mRNA, complete cds | AY672636 | 1.00E-78 | *Heterodera glycines* | 3.4 |
|  | Hg3J2-CT24 | JZ532494 | 395 | Hypothetical lipoprotein | YP_001210195 | 6.00E-08 | *Dichelobacter nodosus* VCS1703A | 13.6 |
|  | Hg3J2-CT48 | JZ532495 | 394 | 18S small subunit ribosomal RNA gene | FJ040405 | 3.00E-82 | *Heterodera hordecalis* | 2.0 |
|  | Hg3J2-CT16 | JZ532496 | 320 | Cellulase | AAO25506 | 2.00E-38 | *Heterodera glycines* | 2.4 |
|  | Hg4J2-CT26 | JZ532497 | 115 | 18S small subunit ribosomal RNA gene, partial sequence | FJ040405 | 7.00E-54 | *Heterodera hordecalis* isolate 1063 | 2.2 |
| Race 4 at parasitic J2 stage | Hg4J2-04F08 | JZ532498 | 130 | Pectatelyase (pel-4) mRNA, complete cds | HQ123256 | 3.00E-59 | *Heterodera glycines* | 3.8 |
| Race 3 at parasitic J3 stage | Hg3J3-CT5 | JZ532499 | 381 | Hypothetical protein CBG16926 | XM_002645148 | 2.00E-44 | *Caenorhabditis briggsae* | 6.7 |
|  | Hg3J3-01B03 | JZ532500 | 354 | Hypothetical protein CAEBREN_29610 | EGT35291 | 4.00E-24 | *Caenorhabditis brenneri* | 4.7 |
|  | Hg3J3-01C12 | JZ532501 | 287 | Hypothetical protein CAEBREN_05517 | EGT30552 | 6.00E-15 | *Caenorhabditis brenneri* | 4.3 |
|  | Hg3J3-01G05 | JZ532502 | 383 | Casein kinase I isoform epsilon | ADY43888 | 4.00E-62 | *Ascaris suum* | 2.8 |
|  | Hg3J3-CT1 | JZ532503 | 312 | Helicase SKI2W | ADY40367 | 5.00E-10 | *Ascaris suum* | 5.6 |
|  | Hg3J3-04C09 | JZ532504 | 469 | KN motif and ankyrin repeat domain-containing protein 1 | ADY40753 | 2.00E-41 | *Ascaris suum* | 3.3 |
|  | Hg3J3-04D05 | JZ532505 | 277 | DB module family protein | EJW72750 | 3.00E-13 | *Wuchereria bancrofti* | 3.0 |
|  | Hg3J3-05C05 | JZ532506 | 362 | Hypothetical protein CRE_27430 | XP_003114348 | 2.00E-16 | *Caenorhabditis brenneri* | 4.2 |
|  | Hg3J3-07A01 | JZ532507 | 442 | CBR-ACN-1 protein | XP_002644637 | 5.00E-23 | *Caenorhabditis briggsae* | 5.9 |
|  | Hg3J3-07B01 | JZ532508 | 319 | Aspartic protease precursor Hgg-33 | AAL78216 | 8.00E-49 | *Heterodera glycines* | 2.3 |
|  | Hg3J3-07C05 | JZ532509 | 440 | CBR-PQN-32 protein | XP_002631028 | 4.00E-44 | *Caenorhabditis briggsae* | 13.2 |
|  | Hg3J3-07E10 | JZ532510 | 564 | Hypothetical protein LELG_03005 | XP_001526447 | 4.00E-11 | *Lodderomyces elongisporus* NRRL YB-4239 | 6.3 |
|  | Hg3J3-06H09 | JZ532511 | 296 | Serine proteinase | CAA74204 | 1.00E-32 | *Heterodera glycines* | 10.2 |
|  | Hg3J3-07B03 | JZ532512 | 351 | Hypothetical protein CRE_24055 | XP_003099796 | 3.00E-21 | *Caenorhabditis remanei* | 2.5 |
|  | Hg3J3-10F10 | JZ532513 | 523 | Protein F53B1 | NP_508390 | 1.00E-21 | *Caenorhabditis elegans* | 3.8 |
|  | Hg4J3-05D07 | JZ532514 | 479 | Hypothetical protein bm1_50160 | XP_001901500 | 2.00E-11 | *Brugia malayi* | 2.3 |
|  | Hg4J3-07C01 | JZ532515 | 650 | Ubiquitin-conjugating enzyme e2, putative | EGR34490 | 1.00E-34 | *Ichthyophthirius multifiliis* | 2.3 |
|  | Hg4J3-CT55 | JZ532516 | 300 | KN motif and ankyrin repeat domain-containing protein 1 | ADY40753 | 1.00E-17 | *Ascaris suum* | 2.3 |
|  | Hg4J3-11B08 | JZ532517 | 498 | Hypothetical protein CAEBREN_03458 | EGT55741 | 1.00E-06 | *Caenorhabditis brenneri* | 3.4 |
|  | Hg4J3-11B09 | JZ532518 | 585 | Hypothetical protein CBG16926 | XP_002645194 | 1.00E-62 | *Caenorhabditis briggsae* | 6.6 |
|  | Hg4J3-11E01 | JZ532519 | 483 | Cellulose binding protein | ADD62691 | 1.00E-08 | *Heterodera avenae* | 2.1 |
| Race 4 at parasitic J3 stage | Hg4J3-06F09 | JZ532520 | 234 | RNA-binding protein 28 | ADY42627 | 2.00E-14 | *Ascaris suum* | 19.6 |
|  | Hg4J3-07G08 | JZ532521 | 331 | GPN-loop gtpase 3 | ADY47091 | 3.00E-18 | *Ascaris suum* | 2.3 |
|  | Hg4J3-08F06 | JZ532522 | 144 | NADH dehydrogenase subunit 4 | ADK97628 | 2.00E-08 | *Heterodera glycines* | 2.4 |
|  | Hg4J3-08G05 | JZ532523 | 393 | Glutathione S-transferase-1 | ABN64198 | 5.00E-09 | *Meloidogyne incognita* | 2.5 |
|  | Hg4J3-09E10 | JZ532524 | 327 | Protein KIN-4, isoform h | NP_001255556 | 9.00E-06 | *Caenorhabditis elegans* | 2.2 |
|  | Hg4J3-10H04 | JZ532525 | 568 | 17-beta-hydroxysteroid dehydrogenase | ABC59299 | 3.00E-28 | *Heterodera glycines* | 2.3 |
|  | Hg4J3-CT31 | JZ532526 | 277 | Serpin-like protein | ADY45856 | 8.00E-15 | *Ascaris suum* | 2.0 |
|  | Hg4J3-CT80 | JZ532527 | 106 | 18S ribosomal RNA gene, partial sequence | JX406383 | 4.00E-24 | *Rotylenchulus reniformis* | 3.4 |
|  | Hg4J3-CT16 | JZ532528 | 447 | Hypothetical protein CBG01832 | XP_002634261 | 2.00E-14 | *Caenorhabditis briggsae* | 2.0 |
| Race 3 at J4 female stage | Hg3J4-01G02 | JZ532529 | 254 | PREDICTED: similar to AGAP009173-PA | XP_967802 | 4.00E-08 | *Tribolium castaneum* | 2.5 |
|  | Hg3J4-01H11 | JZ532530 | 420 | Hypothetical protein LOAG_04263 | XP_003139848 | 1.00E-25 | *Loa loa* | 6.6 |
|  | Hg3J4-02A03 | JZ532531 | 165 | Protein COL-71 | NP_494562 | 1.00E-06 | *Caenorhabditis elegans* | 384.8 |
|  | Hg3J4-02E05 | JZ532532 | 254 | 14-3-3 protein | XP_001610161 | 8.00E-12 | *Babesia bovis* | 37.4 |
|  | Hg3J4-02H09 | JZ532533 | 451 | Unknown | ADY45733 | 9.00E-08 | *Ascaris suum* | 107.1 |
|  | Hg3J4-04A02 | JZ532534 | 298 | Hypothetical protein BRAFLDRAFT_286684 | XP_002605513 | 8.00E-07 | *Branchiostoma floridae* | 2.7 |
|  | Hg3J4-04G03 | JZ532535 | 215 | Hypothetical protein CBG08058 | XP_002643196 | 2.00E-18 | *Caenorhabditis briggsae* | 4.1 |
|  | Hg3J4-05D11 | JZ532536 | 609 | Protein giant | ADY47733 | 2.00E-10 | *Ascaris suum* | 7.4 |
|  | Hg3J4-06B04 | JZ532537 | 464 | Hypothetical protein WUBG_07757, partial | EJW81335 | 2.00E-10 | *Wuchereria bancrofti* | 131.1 |
|  | Hg3J4-06B10 | JZ532538 | 436 | Hypothetical protein CAEBREN_04192 | EGT40044 | 7.00E-35 | *Caenorhabditis brenneri* | 3.3 |
|  | Hg3J4-06E03 | JZ532539 | 134 | Hypothetical protein CAEBREN_03300 | EGT45002 | 9.00E-06 | *Caenorhabditis brenneri* | 28.6 |
|  | Hg3J4-06F04 | JZ532540 | 207 | PREDICTED: RING-box protein 2 | XP_003942632 | 2.00E-13 | *Saimiri boliviensis* | 2.5 |
|  | Hg3J4-06G09 | JZ532541 | 182 | *Eristalis tenax* partial mRNA for hypothetical protein (ORF1), isolate 3 | AM706411 | 2.00E-06 | *Eristalis tenax* | 3.2 |
|  | Hg3J4-07C06 | JZ532542 | 129 | 28S ribosomal RNA gene, partial sequence | JQ040527 | 4.00E-65 | *Heterodera schachtii* | 2.7 |
|  | Hg3J4-07C08 | JZ532543 | 224 | Protein ASP-2, isoform a | NP_505384 | 8.00E-10 | *Caenorhabditis elegans* | 2.2 |
|  | Hg3J4-07H09 | JZ532544 | 438 | Serine carboxypeptidase F41C3.5 precursor | XP_001900088 | 9.00E-19 | *Brugia malayi* | 2.8 |
|  | Hg3J4-09B07 | JZ532545 | 415 | Protein C14B9.3 | NP_498771 | 6.00E-18 | *Caenorhabditis elegans* | 7.7 |
|  | Hg3J4-09E07 | JZ532546 | 220 | Unnamed protein product | CCD58743 | 1.00E-23 | *Schistosoma mansoni* | 2.0 |
|  | Hg3J4-09E09 | JZ532547 | 177 | Hypothetical protein WUBG_11461 | EJW77631 | 1.00E-07 | *Wuchereria bancrofti* | 2.7 |
|  | Hg3J4-09G01 | JZ532548 | 431 | Putative calcium-independent phospholipase A2 isoform a | AAT06310 | 5.00E-09 | *Dictyocaulus viviparus* | 8.6 |
|  | Hg3J4-CT10 | JZ532549 | 567 | Serine proteinase | CAA74204 | 4.00E-87 | *Heterodera glycines* | 3.9 |
|  | Hg3J4-CT13 | JZ532550 | 329 | Hypothetical protein CRE_16678 | XP_003106779 | 4.00E-08 | *Caenorhabditis remanei* | 7.2 |
|  | Hg3J4-CT17 | JZ532551 | 257 | Hypothetical protein CBG13677 | XP_002632455 | 4.00E-11 | *Caenorhabditis briggsae* | 3.5 |
|  | Hg3J4-CT2 | JZ532552 | 496 | Ankyrin repeat domain-containing protein 13C-B | EKC27424 | 2.00E-07 | *Crassostrea gigas* | 12.4 |
|  | Hg3J4-CT21 | JZ532553 | 291 | Protein T21D12.12 | NP_499892 | 2.00E-16 | *Caenorhabditis elegans* | 22.2 |
|  | Hg3J4-CT28 | JZ532554 | 325 | Hypothetical protein WUBG_10861 | EJW78230 | 7.00E-08 | *Wuchereria bancrofti* | 3.9 |
|  | Hg3J4-CT30 | JZ532555 | 317 | Hypothetical protein OXYTRI_13058 | EJY66653 | 5.00E-17 | *Oxytricha trifallax* | 3.8 |
|  | Hg3J4-CT32 | JZ532556 | 244 | Col-1 gene for putative cuticular collagen, exons 1-4 | AJ277425 | 1.00E-11 | *Globoderapallida* | 353.1 |
|  | Hg3J4-CT35 | JZ532557 | 518 | Protein T06D8.10 | NP_496407 | 1.00E-57 | *Caenorhabditis elegans* | 21.6 |
|  | Hg3J4-CT36 | JZ532558 | 426 | Warthog protein 4 | ADY46216 | 1.00E-12 | *Ascaris suum* | 4.7 |
|  | Hg3J4-CT48 | JZ532559 | 481 | UDP-Gal:betaGlcNAc beta 1,4- galactosyltransferase polypeptide 1 | XP_002130226 | 1.00E-13 | *Ciona intestinalis* | 26.1 |
|  | Hg3J4-CT54 | JZ532560 | 194 | Clone Hg-Con2 unknown mRNA | AY853178 | 2.00E-99 | *Heterodera glycines* | 34.2 |
|  | Hg3J4-CT55 | JZ532561 | 461 | Unknown | ADY45733 | 7.00E-19 | *Ascaris suum* | 210.7 |
|  | Hg3J4-CT26 | JZ532562 | 293 | Polyubiquitin | CAL30085 | 4.00E-19 | *Globodera pallida* | 2.0 |
|  | Hg3J4-CT39 | JZ532563 | 519 | Hypothetical protein Bm1_17870 | XP_001895031 | 1.00E-18 | *Brugia malayi* | 2.0 |
|  | Hg4J4-CT42 | JZ532564 | 514 | Transcript antisense to ribosomal RNA protein | EGC42647 | 2.00E-13 | *Ajellomyces capsulatus* H88 | 4.5 |
|  | Hg4J4-02C07 | JZ532565 | 227 | Unnamed protein product | CCD58743 | 2.00E-24 | *Schistoso mamansoni* | 2.5 |
|  | Hg4J4-02E07 | JZ532566 | 492 | CBN-SQT-1 protein | EGT44965 | 1.00E-12 | *Caenorhabditis brenneri* | 4.3 |
|  | Hg4J4-03C06 | JZ532567 | 223 | Col-1 gene for putative cuticular collagen, exons 1-4 | AJ277425 | 3.00E-11 | *Globoderapallida* | 2.0 |
|  | Hg4J4-05D02 | JZ532568 | 451 | Neprilysin-1 | ADY42124 | 1.00E-58 | *Ascaris suum* | 2.0 |
|  | Hg4J4-06C04 | JZ532569 | 341 | Fructose-1,6-bisphosphatase 1 | ADY48557 | 4.00E-22 | *Ascaris suum* | 2.1 |
|  | Hg4J4-06F02 | JZ532570 | 295 | Ornithine decarboxylase antizyme | Q9NHZ4 | 5.00E-07 | *Pristionchus pacificus* | 2.1 |
| Race 4 at J4 female stage | Hg4J4-01F11 | JZ532571 | 255 | PREDICTED: similar to CG32495 | XP_002165112 | 7.00E-13 | *Hydra magnipapillata* | 155.7 |
|  | Hg4J4-05D07 | JZ532572 | 290 | Mannitol dehydrogenase domain protein | ZP_09290804 | 3.00E-06 | *Mesorhizobium alhagi* CCNWXJ12-2 | 8.5 |
|  | Hg4J4-06A04 | JZ532573 | 239 | CBN-COL-138 protein | EGT42039 | 8.00E-07 | *Caenorhabditis brenneri* | 2.6 |
|  | Hg4J4-06B03 | JZ532574 | 215 | Neurobeachin | EKC35427 | 4.00E-18 | *Crassostrea gigas* | 2.6 |
|  | Hg4J4-06D05 | JZ532575 | 384 | Hypothetical protein CBG01611 | XP_002634069 | 1.00E-30 | *Caenorhabditis briggsae* | 2.0 |
|  | Hg4J4-06G09 | JZ532576 | 355 | Hypothetical protein | AEO33083 | 3.00E-12 | *Amblyomma maculatum* | 2.4 |
|  | Hg4J4-07E04 | JZ532577 | 133 | NADH dehydrogenase subunit 1 | ADK97620 | 4.00E-07 | *Heterodera glycines* | 2.5 |
|  | Hg4J4-07F07 | JZ532578 | 687 | N-acetylgalactosamine kinase-like | AFJ49288 | 5.00E-28 | *Crotalus adamanteus* | 2.5 |
|  | Hg4J4-09C10 | JZ532579 | 245 | Glutathione S-transferase-1 | ABN64198 | 8.00E-13 | *Meloidogyne incognita* | 10.7 |
|  | Hg4J4-09D03 | JZ532580 | 221 | Chorismate mutase | AAO19577 | 9.00E-35 | *Heterodera glycines* | 39.2 |
|  | Hg4J4-CT26 | JZ532581 | 442 | Putative gland protein G11A06 | AAP30754 | 4.00E-37 | *Heterodera glycines* | 18.6 |
|  | Hg4J4-CT33 | JZ532582 | 388 | CLAVATA3/ESR (CLE)-related protein 2 | Q86RQ1 | 3.00E-29 | *Heterodera glycines* | 498.0 |
|  | Hg4J4-CT53 | JZ532583 | 427 | Hypothetical protein CAEBREN_28043 | EGT36283 | 9.00E-19 | *Caenorhabditis brenneri* | 5.6 |

^a^ The expression level was analyzed with the 2^-ΔΔCt^ method using the GAPDH gene as an internal reference gene for normalization. For race 3-enriched genes, the calibrator was the transcript level of the corresponding genes in race 4 at the same developmental stage and reverse for race 4-enriched genes.
